# Appropriate Intervention Diets to Manage Type 2 Diabetes Mellitus Among Adults in Australia: A Systematic Review

**DOI:** 10.7759/cureus.95899

**Published:** 2025-11-01

**Authors:** Abdullah S Almarhabi, Ali E Alkhari, Ahmed Binmahfoz, Adel O Tawhari, Sultan A Almehwari

**Affiliations:** 1 Department of Public Health, Faculty of Health, Queensland University of Technology, Brisbane, AUS; 2 Department of Health Programs, Ministry of Health, Al-Qunfudhah, SAU; 3 Department of Public Health, Umm Al-Qura University, Al-Leith, SAU; 4 Department of Laboratory Sciences, Eastern Health Cluster, Dammam, SAU; 5 Department of Family and Community Medicine, King Fahad Armed Forces Hospital, Jeddah, SAU

**Keywords:** australia, diabetes mellitus type 2, diet, intervention, systematic review

## Abstract

Type 2 diabetes mellitus (T2DM) is a chronic metabolic disorder characterised by insulin resistance and hyperglycaemia, contributing to significant morbidity and mortality worldwide, including in Australia. This systematic review aimed to explore the most appropriate dietary interventions for managing T2DM among Australian adults. Following the Preferred Reporting Items for Systematic Reviews and Meta-Analyses (PRISMA) guidelines, databases including PubMed, Medline, Embase, and Google Scholar were searched for studies published between 2010 and 2025. Eligible studies involved Australian adults with diagnosed T2DM and evaluated the effects of low-carbohydrate, Mediterranean, vegetarian, vegan, or intermittent fasting (IF) diets on glycaemic control, weight management, and diabetes-related complications.

Thirteen studies, comprising randomised controlled trials, meta-analyses, and systematic reviews, met the inclusion criteria. Findings indicated that low-carbohydrate diets effectively improved glycaemic control and reduced HbA1c and fasting glucose levels. The Mediterranean diet showed strong evidence for improving glycaemic parameters, enhancing cardiovascular health, and reducing insulin resistance. Vegetarian and vegan diets improved insulin sensitivity and glycaemic control due to high intake of fibre, antioxidants, and unsaturated fats, while IF demonstrated modest benefits in HbA1c reduction and weight loss.

Overall, all five dietary interventions were beneficial for T2DM management, but the Mediterranean and low-carbohydrate diets showed the most consistent and sustained improvements in metabolic outcomes. These findings support integrating dietary modification into diabetes care programs in Australia. Further research is warranted to determine long-term adherence, safety, and the optimal dietary approach for individualised management of T2DM.

## Introduction and background

Type 2 diabetes mellitus (T2DM) is a typical form of diabetes and is considered among the most prevalent diseases worldwide. T2DM consists of a variety of physical dysfunctions predominantly characterised by hyperglycaemia and caused by a combination of resistance to the action of insulin and/or the inadequate secretion of insulin [1]. As a result, the glucose produced by the body fails to enter cells, effectively resulting in high levels of blood glucose as it is discharged into the bloodstream. As the levels of blood sugar in the body increase, insulin-producing beta cells are forced to produce more insulin. Over time, these cells become increasingly impaired, subsequently failing to produce adequate insulin to satisfy the needs of the body [1].

Zheng et al. noted that, statistically, in 2015, one in 11 adults aged between 20 and 79 years old (or approximately 415 million adults) worldwide had diabetes mellitus [1]. Moreover, it was estimated that the population of adults with diabetes mellitus will hit 642 million by 2040 [1]. In Australia, the burden of T2DM is equally enormous. About one million Australian adults (5%) had T2DM in 2014 to 2015 based on self-reported data from the National Health Survey [2]. According to Schofield et al., besides the cost of treating the condition, diabetes further costs Australia nearly $A2.1 billion due to its limiting impact on productive life years [3]. Still, the current estimates of diabetes may be underrepresented because many cases often go unreported in self-reported surveys.

T2DM produces disabling and life-threatening complications. For example, Wu et al. argue that it increases the risk of heart disease, hypertension, stroke, and atherosclerosis [4] . It also causes nerve damage, hearing impairment, kidney damage, slow healing, eye damage, sleep apnoea, various skin conditions, and Alzheimer’s disease.

Evidently, effective interventions should be implemented to address T2DM. Many studies have demonstrated that the condition can be managed through lifestyle changes [5-8]. Foremost, being overweight has been established as a critical risk factor for T2DM [1]. Consequently, dietary management has been considered as one of the beneficial interventions for managing T2DM among adults [5,6]. For example, a study by Mottalib et al. examined the influence of dietary management on haemoglobin A1c (HbA1c) level and certain cardiovascular disease risk factors (e.g., waist circumference and body fat percentage) in obese and overweight patients with T2DM and found that structured dietary management improves glycaemia and reduces cardiovascular disease risk factors [9]. However, since most studies to date have drawn mixed conclusions regarding the effectiveness of various dietary guidelines in producing the desired outcomes relating to crucial components of managing T2DM (such as weight maintenance, improvement of glycaemic control, and reduction of the risks associated with macrovascular and microvascular complications) there remains a need to establish an optimal dietary recommendation for Australian adults.

The Mediterranean diet is a predominantly plant-based dietary pattern that comes from the traditional eating habits of countries bordering the Mediterranean Sea [10]. It emphasises high consumption of fruits, vegetables, whole grains, legumes, nuts, and olive oil; moderate intake of fish, poultry, and dairy products; and limited consumption of red meat and processed foods [10]. 

T2DM has a complex aetiology that consists of both reversible and irreversible factors. The former includes smoking, diet, and physical activity, while the latter entails aspects such as ethnicity, race, age, and genetics [11]. The purpose of this study was to assess various studies on dietary recommendations for the management of T2DM to establish the most appropriate one for Australian adults. Also, provides multiple health care stakeholders involved in diabetes care (e.g., health facilities, health care providers, and relevant agencies) with synthesised information useful in encouraging patients and the public to understand and acknowledge the importance of various dietary guidelines in the management of T2DM to ensure improved quality of life.

Moreover, explores the overall impact of different dietary intakes on the management of T2DM among adults within Australia. Accordingly, it includes a discussion of the effect of dietary management of hyperglycaemia, establishes the effect of dietary management on weight, and assesses the protective role of dietary management against T2DM complications.

This study aimed to conduct a systematic review to explore the appropriateness of different dietary intakes, namely, low-carbohydrate, Mediterranean, vegetarian, vegan, and intermittent fasting (IF), on the management of T2DM amongst adults in Australia. Consequently, it aims to evaluate whether any dietary management styles possess a level of efficacy that warrants their use in addressing T2DM.

## Review

Methods

This systematic review was conducted based on Preferred Reporting Items for Systematic Reviews and Meta-Analyses (PRISMA) guidelines for systematic reviews to ensure comprehensive reporting and transparency of published studies that have explored the relationship between dietary management and T2DM [12].

Inclusion and Exclusion Criteria

The systematic review encompassed longitudinal studies and randomised controlled trials. It includes quasi-experimental studies and systematic reviews in addition to meta-analysis. Eligibility criteria include participants who are Australian adults with a positive diagnosis of T2DM, and evaluation of dietary management consisting of diets such as low-carbohydrate, Mediterranean, vegetarian, vegan and intermittent fasting. The studies also have details on one or more of the following research outcomes: blood glucose levels, weight and diabetes complications.

Articles published earlier than 2010 were not included in the analysis. The use of publishing dates as an exclusion factor ensures the data generated are current and relevant in responding to contemporary diabetes issues. Articles published in languages other than English were excluded. Also, published papers had been excluded if the participants had no diagnosis of T2DM or diagnosis was not made by qualified clinical personnel.

Information Sources and Search Strategy

A comprehensive literature search was performed across PubMed, Medline (Ovid), Embase, and Google Scholar. Additional sources included reference lists of included articles and grey literature to minimise publication bias. The literature search was conducted in January 2025, with articles to be included having a publishing date no earlier than January 2010. The search was restricted to English-language publications involving adult participants (≥18 years) diagnosed with T2DM. 

The search strategy combined both Medical Subject Headings (MeSH) and free-text terms to capture all relevant literature. Core keywords included: “Type 2 Diabetes Mellitus,” “T2DM,” “dietary intervention,” “low-carbohydrate diet,” “Mediterranean diet,” “vegetarian diet,” “vegan diet,” “intermittent fasting,” “glycaemic control,” “HbA1c,” “insulin resistance,” and “Australia.” Boolean operators AND and OR were applied to combine terms appropriately.

Additional filters were applied to limit results to human studies, clinical trials, systematic reviews, and meta-analyses. To ensure comprehensiveness, reference lists of all included studies and relevant review articles were manually screened to identify further eligible publications. Grey literature, including dissertations and conference abstracts, was also examined through Google Scholar and ProQuest to minimise publication bias.

Study Selection

According to Moher et al., the study selection should be systematic to ensure all relevant articles are captured [13]. The first phase consists of identifying all studies generated from the database search. The second involves screening, during which all duplicates will be removed. The third phase consists of testing the articles for eligibility based on the inclusion and exclusion criteria. All exclusions were accompanied by an explanation of why the study failed to meet the inclusion guidelines.

First, two reviewers scan the title of the article and the abstract; after excluding irrelevant articles, they look at the entire article. In the case of disagreement in study selection, either the reviewers will resolve the issue through discussion, or a third author will be involved to arbitrate.

Data Collection Process

Data were extracted independently by two reviewers using a standardised extraction form adapted from the Joanna Briggs Institute (JBI) Manual for Evidence Synthesis [14]. Extracted data included study characteristics (author, year, country, design, sample size, intervention, duration), outcomes (HbA1c, fasting glucose, weight, lipid profile, and complications), and main findings.

Risk of Bias Assessment

Risk of bias for randomised trials was assessed using the Cochrane Risk of Bias 2 (RoB 2) tool [15]. For systematic reviews and observational studies, the JBI critical appraisal checklists were applied [16]. Any disagreements were resolved by consensus.

Data Synthesis

Given heterogeneity in interventions and outcome measures, a narrative synthesis summarised findings across comparable dietary interventions. Results were categorised by dietary type (low-carbohydrate, Mediterranean, vegetarian/vegan, and intermittent fasting) and reported with consideration to clinical and methodological diversity.

Results

**Figure 1:**
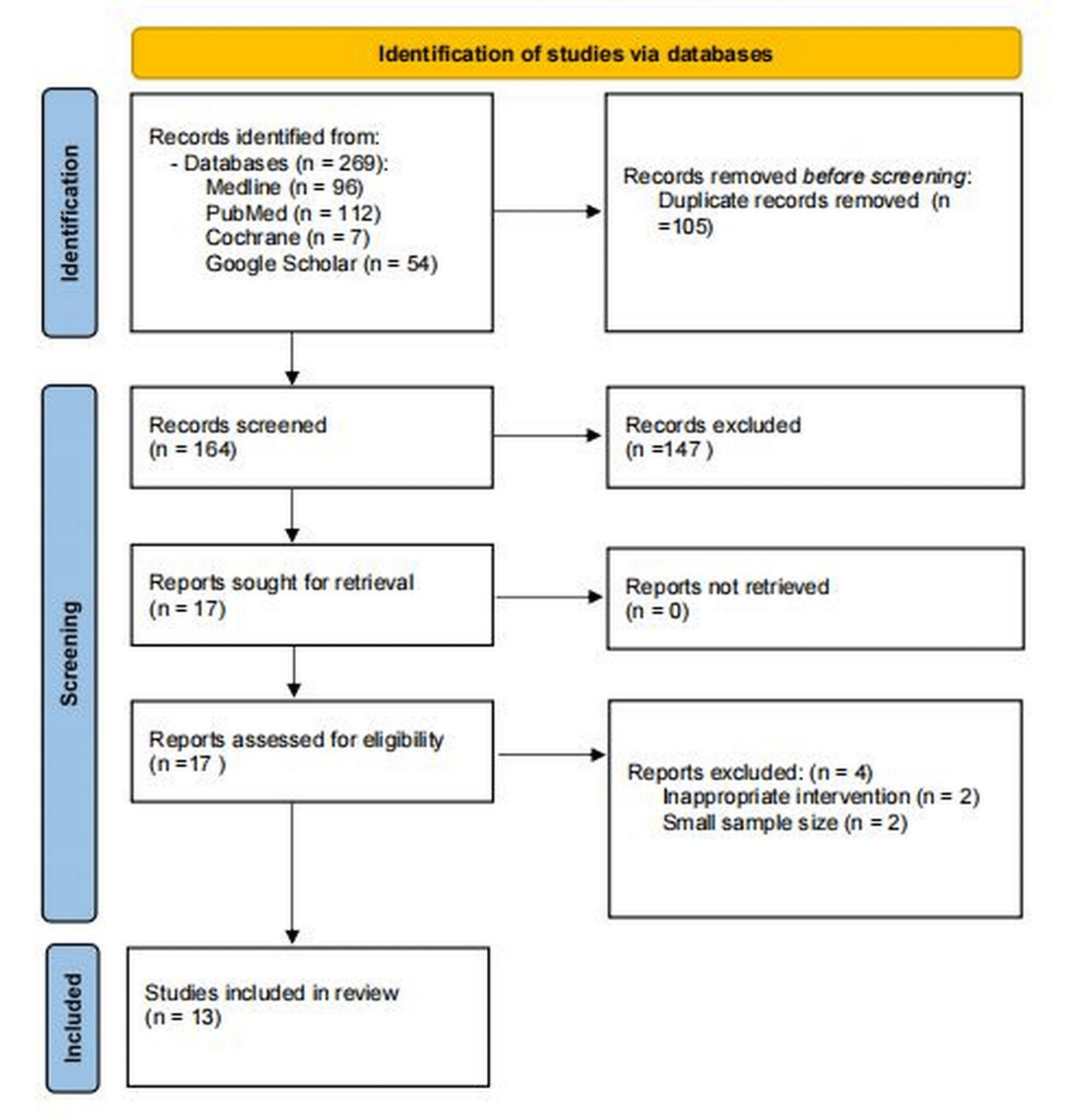
Show PRISMA flow chart for study selection PRISMA: Preferred Reporting Items for Systematic Reviews and Meta-Analyses

During the first search, 269 records were found. After removing duplicates, a total of 164 records remained. The titles and abstracts of all remaining articles were examined to establish which were relevant to the study topic or research question. A total of 147 records were excluded due to issues such as irrelevant target populations, irrelevant outcomes, and the presence of diseases other than T2DM. A total of 17 full-text articles were assessed for eligibility. Four were excluded due to small sample size, inappropriate intervention, wrong setting or wrong dosage. The records excluded following the eligibility review were subtracted from the total count of eligible articles. In total, 13 studies were included in meta-analyses. Figure 1* *shows the PRISMA flow diagram.

The project focused only on quantitative synthesis, so no qualitative synthesis was included. Finally, the Joanna Briggs Institute checklist was used to evaluate the quality of the studies included by particularly assessing what counts as evidence as well as the method used in synthesising the various types of evidence [16].

Characteristics and Main Outcomes of Each Study

The 13 included articles were systematic reviews, meta-analyses, randomised trials, a systematic review of randomised trials, and translational studies (Table 1).

**Table 1:** Summary of included studies characteristics and outcomes T2DM: Type 2 diabetes mellitus; HbA1c: glycated hemoglobin; CVD: cardiovascular Disease; BMI: body mass index; RCT: randomised controlled trial; TRE: time-restricted eating; DIET: conventional dietary intervention; CI: confidence interval; TDR: total diet replacement; LC: low-carbohydrate; HC: high-carbohydrate; SD: standard deviation

Author(s)	Year	Study design	Sample size	Key findings
Ajala et al. [17]	2013	Systematic review and meta-analysis	3,073	Reduction of blood glucose concentration, including loss of weight and improvement of the lipid profile, minimises the chances of cardiovascular risks, especially in individuals with T2DM
Burch et al. [18]	2018	Systematic review	13,662	Focused on five main food groups: grains, fruits, meat alternatives, dairy and vegetables. This literature synthesis established that adults with T2DM are not compliant with the recommended food group intake.
Carter et al. [19]	2018	Randomised noninferiority trial	137	This trial was based on the knowledge that intermittent energy restriction can be an alternative method of losing weight. The primary outcome was alteration in HbA1c level, while the second result was loss of weight
Clifton et al. [20]	2016	A translational study	43	The outcomes included “a reduction of both HbA1c and fasting glucose among the low-carbohydrate diet group.
Esposito et al. [21]	2015	Systematic review and meta-analysis	13 studies	Adults with or at risk of T2DM were subjected to dietary patterns considered the Mediterranean diet, and it was established that this dietary approach improved their glycaemic control and CVD risk factors.
Georgoulis et al. [22]	2014	Systematic review and meta-analysis	4,365	Mediterranean diet provides several benefits for the management and control of T2DM among adult patients. Key among them were better glycaemic control and increased weight management
Papamichou et al. [23]	2019	Systematic review of randomised clinical trials	20 studies	Significant outcomes include improved glycaemic control, reduced HbA1c level, greater body mass loss, and improved insulin sensitivity from both vegan and vegetarian diets
Sleiman et al. [24]	2015	Systematic review	24 studies	The study established that a Mediterranean diet has favourable effects on CVD and glycaemic control.
Tay et al. [25]	2014	Randomised controlled trial	115	Focused on “comparing the effects of a very low-carbohydrate, high-unsaturated/low-saturated fat diet (LC) with those of a high-unrefined carbohydrate, low-fat diet (HC) on glycaemic control and cardiovascular disease (CVD) risk factors in type 2 diabetes. Results showed that both diets led to useful improvements in various CVD risk factors and glycaemic control.
Snorgaard et al. [26]	2017	Systematic review and meta-analysis	1,376	The review revealed that restricting carbohydrates lowers blood glucose and HbA1c levels.
Dening et al. [27]	2023	Randomised controlled trial	87	Improved significantly in the intervention arm: weight −3.26 kg (p<0.0001), BMI −1.11 kg/m² (p<0.0001) and reduced anti-glycaemic medication requirements (mean change −0.40 units; p<0.0001). Effect sizes were large (Cohen’s d > 0.8). (T2Diet RCT — remote, 16-week).
Parr et al. [28]	2024	Randomised controlled trial	51	Both groups achieved statistically significant HbA1c reductions (overall P=0.002): TRE −0.4% (≈ −5 mmol/mol) and DIET −0.3% (≈ −4 mmol/mol). Non-inferiority testing (margin 0.3% HbA1c) showed TRE was non-inferior to DIET (difference −0.11%, 95% CI −0.50 to 0.28).
Hocking et al. [29]	2023	Open-label, primary-care translation of the DiRECT TDR protocol	155	The authors conclude that in an Australian primary-care setting, a structured low-energy TDR programme achieved remission for approximately one in two participants at 12 months; they reported high clinical effectiveness but highlighted the need to monitor safety and support long-term weight maintenance.

Ajala et al. was a “systematic review and meta-analysis of different dietary approaches for managing type 2 diabetes” [17]. This study was based on evidence suggesting that a “reduction of blood glucose concentration, including loss of weight and improvement of the lipid profile, minimises chances of cardiovascular risks, especially in individuals with T2DM”. Specifically, it conducted searches of different databases, such as Google Scholar, PubMed and Embase, to evaluate the “effect of different diets (such as low-carbohydrate, high-fibre, low-protein diets, low-glycaemic index, vegan and vegetarian) on lipids, weight loss, and glycaemic control” and established that these foods can effectively “improve different markers of (cardiovascular disease (CVD)) risk among the target population”.

Burch et al. was a systematic review of “dietary intake by food group of individuals with T2DM” [18] . It screened 11 studies (which were included from the 13,662 publications) obtained from online databases such as Medline and Embase. It focused on five main food groups: grains, fruits, meat alternatives, dairy and vegetables. This literature synthesis established that adults with T2DM are not compliant with the recommended food group intake.

Carter et al. was a randomised noninferiority trial that examined the “effect of intermittent compared with the continuous energy-restricted diet on glycaemic control in patients with type 2 diabetes” [19] . This trial was based on the knowledge that intermittent energy restriction can be an alternative method of losing weight. A total of 137 participants with T2DM were used in this study between 2015 and 2017 at the University of South Australia. The primary outcome was alteration in HbA1c level, while the second result was loss of weight.

Clifton et al. was a translational study that focused on establishing whether “a piece of simple advice to make modest reductions in carbohydrates is effective in clinical practice given that intensive interventions with low carbohydrate diets reduce HbA1c in T2DM patients” [20]. The design included a random selection of 43 individuals with T2D who were subjected to “two short education sessions for six months on how to minimise the intake of carbohydrate by approximately 25%.” The outcomes included “a reduction of both HbA1c and fasting glucose among the low-carbohydrate diet group.”

Esposito et al. was a systematic review with meta-analysis. It focused on summarising existing “evidence about the efficacy of a Mediterranean diet on the management of T2D and prediabetic states” [21]. The design of this study entails a comprehensive “review of both meta-analyses and [RCTs] that compared the Mediterranean diet with a controlled diet regarding the treatment of T2D and prediabetic conditions.” Accordingly, “adults with or at risk of T2DM were subjected to dietary patterns considered the Mediterranean diet”, and it was established that this dietary approach improved their glycaemic control and CVD risk factors.

Georgoulis et al. was a review that examined “existing scientific knowledge on the relationship between a Mediterranean diet and T2DM” [22]. It reviewed RCTs and prospective cohort studies to establish that a Mediterranean diet provides several benefits for the management and control of T2DM among adult patients. Key among them were better glycaemic control and increased weight management.

Papamichou et al. was a systematic review of randomised clinical trials [23]. This study aimed at examining “evidence from published studies on the effectiveness of diets, such as low-carbohydrate, vegan, Mediterranean, intermittent fasting, and vegetarian, on the control and management of diabetes for at least six months”. It adopted the PRISMA design and searched several databases. Significant outcomes include “improved glycaemic control, reduced HbA1c level, greater body loss, and improved insulin sensitivity from both vegan and vegetarian diets”.

Sleiman et al. was a systematic review of the “effect of the Mediterranean diet on diabetes control and cardiovascular risk modification” [24]. It was based on the knowledge that T2DM has been traditionally controlled through modification of lifestyle, particularly change of diet and engagement in physical activity. A comprehensive search was conducted on popular electronic databases, such as Google Scholar, Cochrane, Medline, and PubMed. The study established that a Mediterranean diet has favourable effects on CVD and glycaemic control.

Tay et al. was a randomised trial [25]. It focused on “comparing the effects of a very low-carbohydrate, high-unsaturated/low-saturated fat diet (LC) with those of a high-unrefined carbohydrate, low-fat diet (HC) on glycaemic control and cardiovascular disease (CVD) risk factors in type 2 diabetes (T2DM)”. It subjected “115 obese adults with T2DM (between 51 and 65 years old) to LC diet combined with structured exercise for 24 weeks”. Results showed that both diets led to useful improvements of various CVD risk factors and glycaemic control.

Snorgaard et al. was a “systematic review and meta-analysis of dietary carbohydrate restriction in patients with type 2 diabetes” [26]. Sources published in the Cochrane, Medline and Embase databases between 2004 and 2014 were systematically reviewed for dietary guidelines, RCTs and meta-analyses evaluating the outcomes in terms of cholesterol, HbA1c level, weight and quality of life. The review revealed that restricting carbohydrates lowers blood glucose and HbA1c levels.

Dening et al., who conducted a randomised trial, found that at 16 weeks the web-based low-carbohydrate programme plus standard care produced a between-group HbA1c reduction of −0.65% (95% CI −0.99 to −0.30; p<0.0001) versus standard care alone [27]. Secondary outcomes improved significantly in the intervention arm: weight −3.26 kg (p<0.0001), BMI −1.11 kg/m² (p<0.0001) and reduced anti-glycaemic medication requirements (mean change −0.40 units; p<0.0001). Effect sizes were large (Cohen’s d > 0.8). (T2Diet RCT - remote, 16-week).

Parr et al. conducted a study in which 51 overweight/obese adults with T2D (HbA1c ≥6.5%) were randomised to TRE (10:00-19:00) or dietitian-led individualised guidance for six months; 43 completed the intervention [28]. Both groups achieved statistically significant HbA1c reductions (overall P=0.002): TRE −0.4% (≈ −5 mmol/mol) and DIET −0.3% (≈ −4 mmol/mol). Non-inferiority testing (margin 0.3% HbA1c) showed TRE was non-inferior to DIET (difference −0.11%, 95% CI −0.50 to 0.28). Body mass decreased in both groups (TRE −1.7 kg; DIET −1.2 kg), driven by an approximate spontaneous energy deficit (~900 kJ/d). Self-reported adherence was higher for TRE (P<0.001) (six-month parallel RCT). 

Hocking et al., who conducted open-label, primary-care translation of the DiRECT TDR protocol among adults 20-65 years with T2DM for duration ≤6 years, BMI >27 kg·m⁻², and not on insulin, found that at 12 months, 86 of 155 participants (56%) achieved diabetes remission (HbA1c <6.5% and no glucose-lowering medication for ≥2 months) [29]. The mean adjusted weight loss at 12 months was −8.1% (95% CI 7.2-9.1). Two serious adverse events requiring hospitalisation were judged related to the intervention. The authors conclude that in an Australian primary-care setting, a structured low-energy TDR programme achieved remission for approximately one in two participants at 12 months; they reported high clinical effectiveness but highlighted the need to monitor safety and support long-term weight maintenance.

Discussion

This review provides evidence that a range of beneficial dietary recommendations exists that are beneficial for adults with T2DM in terms of improving their glycaemic control, weight management, and cardiovascular risk parameters. A low to moderate intake level of carbohydrate diets, for example, has been proven to greatly impact glycaemic control in T2DM patients as compared with foods rich in carbohydrates, even within just the first year of adherence [25]. In other words, the more a T2DM patient adheres to following a restriction in high-carbohydrate food consumption, the better he or she lowers the glucose level. Moreover, besides lowering the HbA1c level in the short term, no diet has yet proven to be superior to low-carbohydrate intake concerning weight management, low-density lipoprotein (LDL) cholesterol regulation, and glycaemic control [25]. In this context, health care providers should feel confident in recommending the restriction of carbohydrate intake to T2DM patients seeking effective weight management and glycaemic control long-term.

The analysis of results from the study by Ajala et al. suggested that modifying the amounts of food nutrients in the diet can effectively improve weight management, glycaemic control, and lipid levels in adults with T2DM [17]. Moreover, diets based on low-carbohydrate intake improve glycaemic control without provoking simultaneous significant improvements in lipoproteins. Restricting the intake of carbohydrates also greatly improves the lipid profile by increasing the HDL, reducing the LDL, and minimising triglyceride levels, respectively [17]. Low-carbohydrate diets are generally low in glycaemic index (GI), which is a way of rating foods based on their glycaemic effect, and are, therefore, effective in lowering the HbAc1 level. Accordingly, such diets provide essential benefits regarding glycaemic control and lipid profile.

Even though the study by Clifton et al. warns against recommending low-carbohydrate foods as default diets on the basis that they do not generate significantly different results beyond the baseline when compared with high-carbohydrate diets with respect to HbA1c changes (partly due to the low number of participants), their findings still offer insight [20]. An analysis of their findings revealed noticeable differences in fasting glucose levels [20]. Therefore, low-carbohydrate intake appears to still reduce HbA1c levels despite a few studies suggesting otherwise. Studies have shown that low-carbohydrate dietary guidelines generate superior weight loss as compared with diets characterised by high carbohydrate and low-fat levels [17]. Despite recording minimal reduction in HbA1c levels, diets characterised by low carbohydrate levels usually ensure the weight loss necessary to assist T2DM patients in further reducing potential cardiovascular risk factors [20].

Besides controlling the intake of carbohydrates, the Mediterranean diet yields greater effectiveness in the improvement of HbA1c among T2DM patients as compared with other diets such as low-GI, low-carbohydrate, and high-protein options [21]. As a result, a Mediterranean eating pattern provides a protective role by minimising the HbA1c level and lessening the fasting glucose level, thereby ensuring a reduced mortality rate and insulin resistance [24]. An evaluation of the available research findings reveals that a Mediterranean dietary recommendation can play a key role in improving cardiovascular risk factors, thereby reducing peripheral artery disease and minimising mortality among T2DM patients [24]. Mediterranean dietary guidelines achieve this goal by improving different cardiovascular risk parameters such as systolic blood pressure, the ratio of total cholesterol to HDL, and the level of triglycerides [24].

The Mediterranean diet is ranked among the most preferable diets for protecting T2DM patients against coronary heart disease. Specifically, long-term adherence to a Mediterranean diet fortified with nuts and olive oils often reduces risks for cardiovascular events such as deaths from cardiovascular diseases, myocardial infarction, or stroke among T2DM patients [21]. Mediterranean diet also helps in decreasing oxidative stress, insulin resistance, and inflammation. In this context, adhering to a Mediterranean diet confers increased chances of remission from metabolic syndrome by half in the long run even though a metabolic syndrome characterizes a prediabetes state [21].

Regarding the protective role of Mediterranean diets against diabetes complications among adults with T2DM in Australia, an analysis of the findings from the study by Georgoulis et al. implied that adherence to a Mediterranean diet is inversely associated with the possibility of being obese in the long term [22]. This interpretation is supported by data from the results section, which suggests that consuming foods rich in compounds such as fruits, vegetables, legumes, and whole grains improves both insulin sensitivity and the capacity of pancreatic β-secretory cells, hence reducing the overall risk of T2DM development among prediabetic adults [22].

Mediterranean foods could be directly responsible for minimising future diabetes incidences by rates between 19% and 23% [21]. Giving these figures a broader viewpoint, the Mediterranean diet can not only be used for managing existing T2DM but is also even more beneficial in preventing diabetes from even developing among those who strictly observe it.

Carter et al. compared intermittent energy restriction to constant energy restriction for controlling glycaemia in patients suffering from T2DM and established a significant change in glycated HbA1c level through severe energy restriction [19]. Even though IF neither yields greater variations in lipid and fasting glucose levels nor significant differences in terms of total medication effect, it, however, reduces HbA1c levels adequately enough to improve glycaemic control. The effects of IF on weight among adults with T2DM are beneficial. For example, besides the primary outcome of reducing haemoglobin levels, IF promotes a considerable loss of weight, which can be equated to 1.75 kilos for a loss in fat mass at 0.75 kilograms [19]. This finding implies that IF can be a safe dietary approach for individuals with diet-controlled T2DM or those using nonhypoglycaemic medications.

An analysis of the existing findings shows that vegetarian and vegan diets are essential in preventing and managing T2DM among adults. Although the study by Papamichou et al. reported no significant variations in glycaemic control, weight management, or lipid control between low-carbon diets and low-fat diets, improvements in these parameters have been observed in conjunction with both vegetarian and vegan diets [23]. Moreover, an analysis of the findings implies that adults suffering from T2DM often cannot strictly adhere to the prescribed intake of carbohydrates but may find it relatively easier to adequately observe vegetarian and vegan diets. The study by Burch et al. found that most of these patients take in less than the recommended serving of fruits, dairy products, vegetables, and grains [18]. Specifically, a critical evaluation of their results indicated that a significant portion of the T2DM patients subscribing to dietary management therapies consume an inadequate amount of fruit, grain, dairy and vegetable servings, while, on the other hand, exceeding the recommended portions of meat or meat alternatives.

Vegan and vegetarian foods are enriched with specific food components that promote insulin sensitivity and improve glycaemic control, such as ‘magnesium, fibre, and antioxidants, as well as those which inhibit glucose absorption, stimulate insulin secretion, reduce hepatic glucose output, and enhance glucose uptake’, they thus provide multiple benefits that make them more fruitful in managing T2DM as compared [with] other contemporary interventions [5].

All vegetarian diets, such as ‘vegetables, unsaturated fats, legumes, fruits, nuts, and whole grains’ have vital functional components that intertwine to manage the symptoms of diabetes [22]. Moreover, while an increased observation of vegan and vegetarian diets improves insulin sensitivity and glycaemic control, the soluble fibre, on the other hand, assists in binding glucose and slowing its absorption rate into the bloodstream [30].

According to McMacken and Shah, plant-based diets, in particular, are beneficial in treating T2DM and limiting both microvascular and macrovascular complications associated with diabetes [5]. The interpretation of this finding is that many foods and nutrient components found in vegan diets play critical roles in preventing and managing T2DM in adults.

In general, the intake of vegetarian and vegan foods as well as adherence to healthy dietary patterns can significantly reduce the chances of developing diabetes. Creating a better awareness regarding the complications involved and required improvements in dietary information, practices, and attitudes is expected to result in improved ways of preventing and managing T2DM.

The present review of the existing literature on dietary approaches for managing T2DM suggests that low-carbohydrate, Mediterranean, vegetarian, vegan, and IF dietary approaches offer effective options for improving glycaemic control, weight management, and various markers of cardiovascular risk in adults with T2DM and should, therefore, be considered in the overall recommendation scheme for preventing and managing T2DM among adults in Australia.

## Conclusions

The review indicates a need to implement effective interventions to address T2DM among adults. Lifestyle changes are regarded as one of the most effective ways of controlling and managing T2DM, since being overweight is a risk factor for both prediabetes and diabetes. Moreover, dietary management is a beneficial intervention for managing T2DM among adults as it produces desired outcomes in crucial components of management, such as weight maintenance, improvement of glycaemic control and reduction of the risks associated with macrovascular and microvascular complications.

However, due to the lack of unanimous agreement across the various studies, there is still a need to establish an optimal dietary recommendation for Australian adults. Also, further research should be carried out to find ways in which these dietary methods can be utilised without causing weight loss among diabetic patients with normal body mass index levels. Moreover, determine how IF can be used effectively among T2DM patients without the risk of hypoglycaemia.
